# Systematic Mapping and Review of Landscape Fire Smoke (LFS) Exposure Impacts on Insects

**DOI:** 10.1093/ee/nvac069

**Published:** 2022-09-21

**Authors:** Yanan Liu, Robert A Francis, Martin J Wooster, Mark J Grosvenor, Su Yan, Gareth Roberts

**Affiliations:** Department of Geography, King’s College London, Bush House, 40 Aldwych, London, WC2B 4BG, UK; Leverhulme Centre for Wildfires, Environment and Society, King’s College London, London WC2R 2LS, UK; Department of Geography, King’s College London, Bush House, 40 Aldwych, London, WC2B 4BG, UK; Department of Geography, King’s College London, Bush House, 40 Aldwych, London, WC2B 4BG, UK; Leverhulme Centre for Wildfires, Environment and Society, King’s College London, London WC2R 2LS, UK; NERC National Centre for Earth Observation, King’s College London, London WC2R 2LS, UK; Department of Geography, King’s College London, Bush House, 40 Aldwych, London, WC2B 4BG, UK; Leverhulme Centre for Wildfires, Environment and Society, King’s College London, London WC2R 2LS, UK; NERC National Centre for Earth Observation, King’s College London, London WC2R 2LS, UK; Department of Electrical and Electronic Engineering, Imperial College London, London SW7 2BX, UK; Geography and Environmental Science, University of Southampton, Southampton, UK

**Keywords:** landscape fire, smoke, insect, behavior, development

## Abstract

Landscape fire activity is changing in many regions because of climate change. Smoke emissions from landscape fires contain many harmful air pollutants, and beyond the potential hazard posed to human health, these also have ecological impacts. Insects play essential roles in most ecosystems worldwide, and some work suggests they may also be sensitive to smoke exposure. There is therefore a need for a comprehensive review of smoke impacts on insects. We systematically reviewed the scientific literature from 1930 to 2022 to synthesize the current state of knowledge of the impacts of smoke exposure from landscape fires on the development, behavior, and mortality of insects. We found: (1) 42 relevant studies that met our criteria, with 29% focused on the United States of America and 19% on Canada; (2) of these, 40 insect species were discussed, all of which were sensitive to smoke pollution; (3) most of the existing research focuses on how insect behavior responds to landscape fire smoke (LFS); (4) species react differently to smoke exposure, with for example some species being attracted to the smoke (e.g., some beetles) while others are repelled (e.g., some bees). This review consolidates the current state of knowledge on how smoke impacts insects and highlights areas that may need further investigation. This is particularly relevant since smoke impacts on insect communities will likely worsen in some areas due to increasing levels of biomass burning resulting from the joint pressures of climate change, land use change, and more intense land management involving fire.

Landscape fires, including wildfires and fires purposely lit for clearing or managing land, are widespread globally, occurring in almost all vegetated biomes worldwide (Fried et al. 2004, 2008; Johnston et al. 2012; Roberts and Wooster 2021). Whilst many biomes may benefit from landscape fires ecologically, not all are well suited to the presence of fire (Keane et al. 2008). In some regions, anthropogenic fires have become too widespread, or are so disparate from the natural regimes that the ecological benefits of fires have diminished (Syphard et al. 2007, 2009). Beyond terrestrial impacts such as the removal of vegetation and the combustion of organic soil, landscape fire has a significant effect on the atmosphere through the smoke released (Keshtkar and Ashbaugh 2007, Gadde et al. 2009, Shi et al. 2014).

This smoke is composed of a mix of gases and airborne particulates, some of which pose risks to normal biological functioning (Erb et al. 2018, Vokina et al. 2019, Sanderfoot et al. 2021). The smoke emissions can affect the air quality locally or even far from the fires (Einfeld et al. 1991, Ward et al. 1992, Streets et al. 2003, Chen et al. 2017, Cascio 2018, Wang et al. 2020, Roberts and Wooster 2021). Whilst most research has focused on the effect of this air pollution on human health (Reid et al. 2016, Roberts and Wooster 2021, Sanderfoot et al. 2021), other animals, including insects, may also be affected. Landscape fires emit thousands of kilograms of carbon into the atmosphere every year – predominantly carbon dioxide (CO_2_), carbon monoxide (CO), and methane (CH_4_) (Jenkins et al. 1992, Andreae and Merlet 2001, Gupta et al. 2004, Gadde et al. 2009, Van der Werf et al. 2010, Zhang et al. 2015, Li et al. 2019, Ravindra et al. 2019). Additionally, nitrogenous gases such as ammonia (NH_3_), nitric oxide (NO), nitrogen dioxide (NO_2_), and nitrous oxide (N_2_O) are also released from fires (Gupta et al. 2004, Oppenheimer et al. 2004, Li et al. 2019, Ravindra et al. 2019). Another group of gaseous emissions emitted in smaller quantities are sulfur-containing gases such as sulfur dioxide (SO_2_) (Gadde et al. 2009; Akagi et al. 2011; Li et al. 2017, 2019), along with smaller quantities of toxic and/or carcinogenic compounds such as hydrogen cyanide (HCH), hydrogen chloride (HCI), benzene (C_6_H_6_), and polycyclic aromatic hydrocarbons (PAH). Certain constituents of the smoke can react to generate other toxic pollutants downwind, such as tropospheric ozone (O_3_) (Jaffe and Wigder 2012, Marlier et al. 2013). These gases may pose a hazard to insects in sufficient concentrations. For example, short term exposure to CO_2_ can act as an anesthetic for *Drosophila melanogaster* (Diptera: Drosophilidae), which leads to a significant decrease in their electroretinogram responses to light stimulation (Stark 1972, Wong et al. 1972, Nicolas and Sillans 1989). CO affects the respiration of insects and causes them to become less active and consume less food (Baker and Wright 1977). NO_2_ has been shown to interfere with the olfactory responses of *Asobara tabida* (Hymenoptera: Braconidae) (Gate et al. 1995). *Drosophila melanogaster*, when exposed to SO_2_ concentrations of around 0.4 ppm in the environment, displayed significantly decreased pupal survival and adult endurance to the polluted environment (Ginevan and Lane 1978).

Particulate matter (PM) is another risk-related concern from LFS (landscape fire smoke), especially those particles of less than 10 microns in diameter (PM_10_) and less than 2.5 microns in diameter (PM_2.5_) (Simoneit 2002, Dhammapala et al. 2007, Broyles 2013). The PM is mainly found in black and organic aerosols, and the PM size distribution is typically skewed strongly toward smaller size particles (Reid et al. 2005, Rissler et al. 2006, Zhang et al. 2011, Roberts and Wooster 2021). The finer PM_2.5_ is regarded as the most significant, health-impacting, and widely transported particulate component of smoke (Johnston et al. 2012, Chen et al. 2017). Fig. 1 shows the fire radiative power density and averaged PM_2.5_ surface level concentration caused by landscape fire in 2017, which Roberts and Wooster (2021) calculated exposed over 65 million people to hazardous PM_2.5_ conditions worldwide. These particulate concentrations are likely to also have direct impacts on insects. For instance, increased concentrations of airborne PM_2.5_ have been linked with shortened lifespans of *Drosophila melanogaster*; in a treatment with an average PM_2.5_ concentration of 80 µg m^−3^, 50% of males and females died after 20 and 21 d respectively, while 50% of flies in filtered air (with an average PM_2.5_ concentration of 4 µg m^−3^) died after 48 and 40 d, respectively (Wang et al. 2017).

**Fig. 1.**
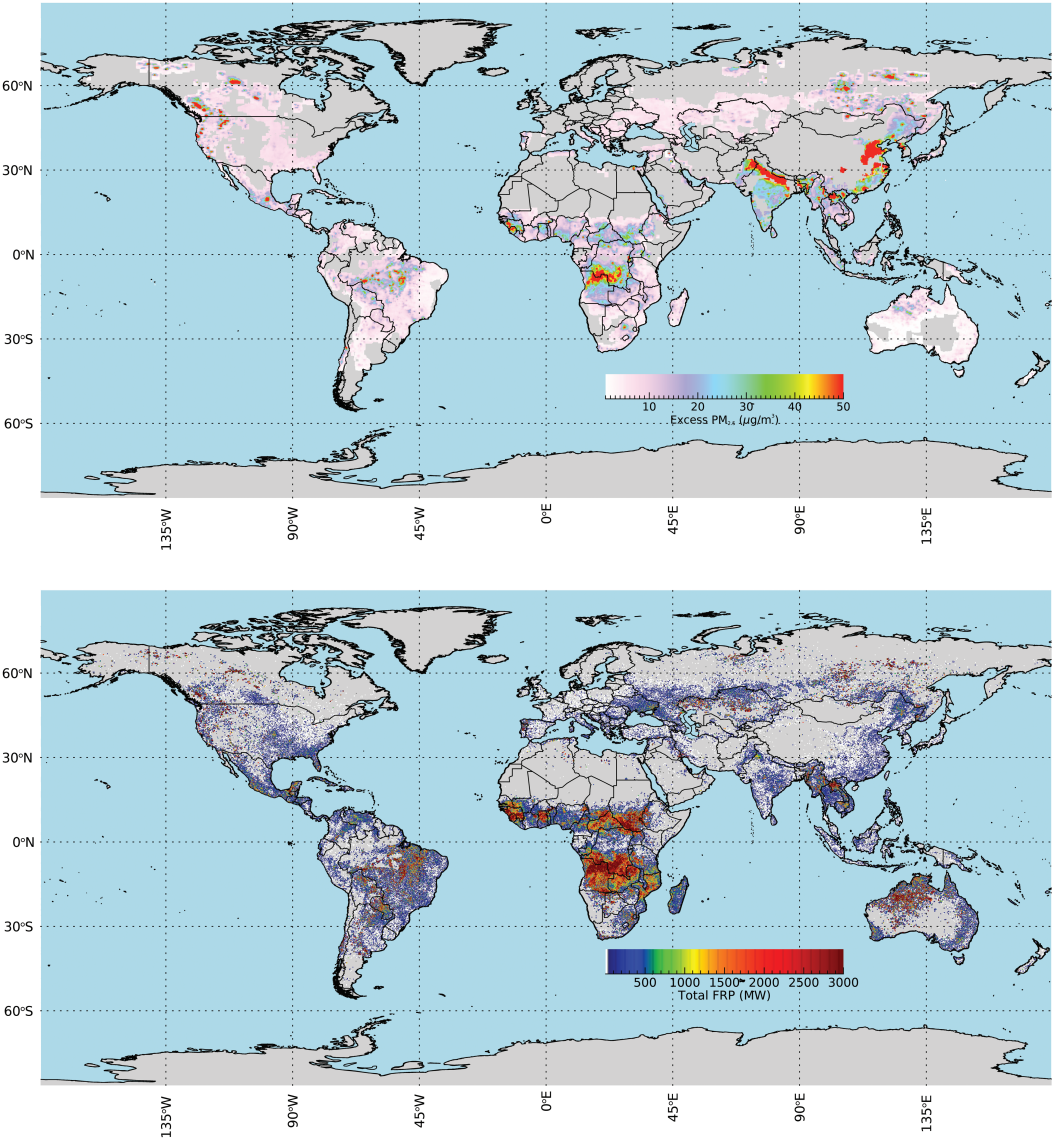
Globally mapped outputs calculated in 2017, with (a) GFAS Fire radiative power (FRP) areal density and (b) the averaged PM_2.5_ surface level concentrations caused by landscape fire-emitted PM_2.5_ (For more information, refer to Roberts and Wooster 2021 for a description of how these data are constructed, and to view longer-term mean plots derived from multiple years).

Previous review articles primarily focused on the effects of LFS on human health (Reid et al. 2016), while the impacts on ecosystems have been less extensively summarized. A comprehensive and systematic synthesis of present research on the effects of LFS on insects is needed to predict the changes in ecosystem services and manage the impact of LFS effectively. This review examines the evidence for the impacts of smoke exposure on insects. Here we focus on LFS and some indoor biomass burning, since the composition of smoke from indoor sources, such as indoor fuelwood burning and incense sticks, is relatively similar to the smoke components from landscape fires (Jetter et al. 2002, Lin et al. 2008, See and Balasubramanian 2011). For instance, in some laboratory work to observe insect response to smoke, smoke emitted from incense coils is used to simulate the haze from forest fires (Tan et al. 2018, Liu et al. 2021). Anthropogenic burning sources such as industrial coal fire, domestic fossil fuel combustion, and traffic engine combustion, were not considered because these commonly occur in anthropogenic settings according to the classification from De Gouw et al. (2004). We used a systematic mapping approach to review the effects of LFS (and other smoke sources that have similar compositions) on insects and we summarized how insects responded to the LFS. Our classification and summary will consolidate the current state of knowledge in this area to facilitate future research on the effects of air pollution on ecosystems and further understand the impacts of climate change on insects.

## Methodology

### Systematic Mapping Methods

Unlike traditional inductive methods for collating information, systematic mapping is a method of organizing, describing, and categorizing available evidence on a specific subject by using an objective and transparent manner to format a usable database and understand knowledge deficiencies (Haddaway et al. 2016, James et al. 2016). We searched papers on PubMed, Web of Science, and Google Scholar using keywords (‘smok*’ and ‘landscape burning’ and ‘insec*’; also with suitable alternatives) to identify those associated with LFS exposure and relevant insect impacts.

Following standard systematic mapping methods, including identification, searching, and screening (James et al. 2016, Berger-Tal et al. 2018), we found 293 articles in PubMed, 296 articles in Web of Science, and 7,070 articles in Google Scholar up to, and including, January 2022. All records obtained were considered, from the earliest articles incorporated in the databases (1930–2022). From these, we removed articles if they described mosquito coil smoke impacts on various types of mosquitoes or focused on tobacco smoke impacts. Mosquito coils are made from base materials such as teak wood and coconut shell powder (Pauluhn 2006). However, they are usually used indoors and overnight to repel mosquitoes by gradually releasing insecticide (Pauluhn 2006, Hogarh et al. 2016). Tobacco is made by drying leaves from tobacco plants, and its smoke contains not only CO_2_, CO, hydrocarbons but also nicotine and aromatic amines (Rodgman and Perfetti 2013). Since the nicotine from tobacco is toxic to many insects, it has been commonly used as a commercial pesticide (Gorrod and Jacob III 1999). We, therefore, did not include those studies because the insecticide chemicals and nicotine released from these sources do not naturally occur in smoke from landscape or domestic wood fires.

After reading abstracts, we scrutinized all records and selected studies that focused on the impacts of real-world LFS and simulated LFS on insects, excluding articles that only studied LFS and were not related to insects, or if they assessed the insect response to stressors not associated with LFS. After accounting for duplicates, we identified 42 unique articles. From the remaining environmental entomology studies (*N* = 42), we extracted the following information from each paper: (1) insect species covered; (2) type and source of smoke pollution; (3) aspects of insect ecology impacted by the pollutant – categorized into development, behavior, and mortality; and (4) geographical location of study.

## Results

### Species of Insect Occurring in Relevant LFS Literature

A total of 42 studies met the criteria for this review, with 40 species of insect studied for their response to smoke polluted conditions. These 40 species were included in twenty-three families and seven orders, including Coleoptera (18 studies), Diptera (12 studies), Hymenoptera (8 studies), Lepidoptera (5 studies), Hemiptera (1 study), Phasmatodea (1 study) and Orthoptera (1 study).

Many species only occurred once in the studies mapped, but some were the foci of several papers. For instance, of the eighteen Coleoptera studies, three focused on *Melanophila acuminata* (Coleoptera: Buprestidae) and other species had two studies individually, including *Monochamus galloprovincialis* (Coleoptera: Cerambycidae), *Rhyzopertha dominica* (Coleoptera: Bostrichidae), *Sericoda bembidioides* (Coleoptera: Carabidae) and *Sitophilus oryzae* (Coleoptera: Curculionidae). Six of twelve Diptera studies referred to *Anopheles gambiae* (Diptera: Culicidae). Five out of eight studies on Hymenoptera focused on *Apis mellifera* (Hymenoptera: Apidae).

### Smoke Types and Sources

**Fig. 2.**
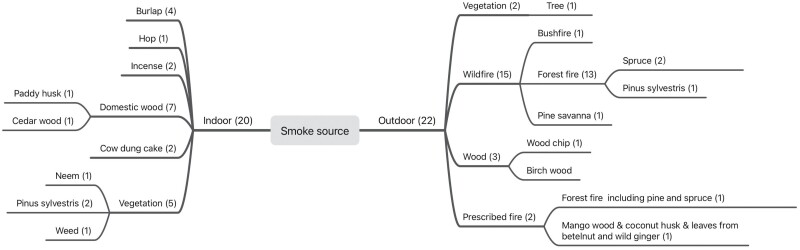
Hierarchy plot showing smoke source classifications collected from 42 papers reviewed, including two general categories (indoor and outdoor) and ten more specific sources, with the number of studies in brackets. Some studies included more than one burning source.

Landscape fires contain various fire types, including forest fires, savanna fires, peat fires, and agricultural fires (Finney 1999, 2004; Keane and Finney 2003; Giglio et al. 2018). A range of smoke sources covered in the literature, is drawn from landscape fires and indoor burning. This study reclassified the reported smoke sources into the two broad indoor and outdoor smoke source categories, with ten specific categories (Fig. 2). Twenty-two studies described the actual landscape fires, of which seventeen articles studied smoke generated from wildfires, and others consisted of wood fire and prescribed fires. Fourteen out of the seventeen wildfire studies focused on forest fires; others included bushfires and savanna fires.

Twenty studies utilized indoor biomass burning to simulate smoke conditions from landscape fires, using fuel such as burlap, cow dung cake, wood, and weeds. Indoor sources in this context include those studies that covered the actual domestic fuel burning activities and biomass sources used in the experimental work to allow more direct measurement of pollutants and particulates. This included where incense coils/sticks were used in the laboratory work, which mainly consist of wood, organic adhesive, and potassium nitrate (Yadav et al. 2020), and therefore, the smoke released by incense has similar components to the smoke from biomass burning (Jetter et al. 2002, Lee and Wang 2004, Lin et al. 2008, Shi et al. 2014). Another two biomass fuels commonly burned indoors are burlap– a woven fabric usually made from the skin of the jute plant and widely used by beekeepers, and hop pellets – which are dried from *Humulus lupulus* (Reilly 1906, Van Cleemput et al. 2009, Gage et al. 2018).

Overall, forty-two papers described eighteen different biomass sources, with one-third of the articles focusing on forest fires (Fig. 2). The dominant emissions in those studies were generically described as ‘smoke’ or ‘volatiles’. Although most papers did not measure the specific gases or particulates produced, they suggested that the smoke emissions from forest fires, vegetation fires, and wood fires contain similar components (Larson and Koenig 1994, Goldammer et al. 2008, Simpson et al. 2011).

### Impacts of Smoke Pollution on Insects

**Fig. 3.**
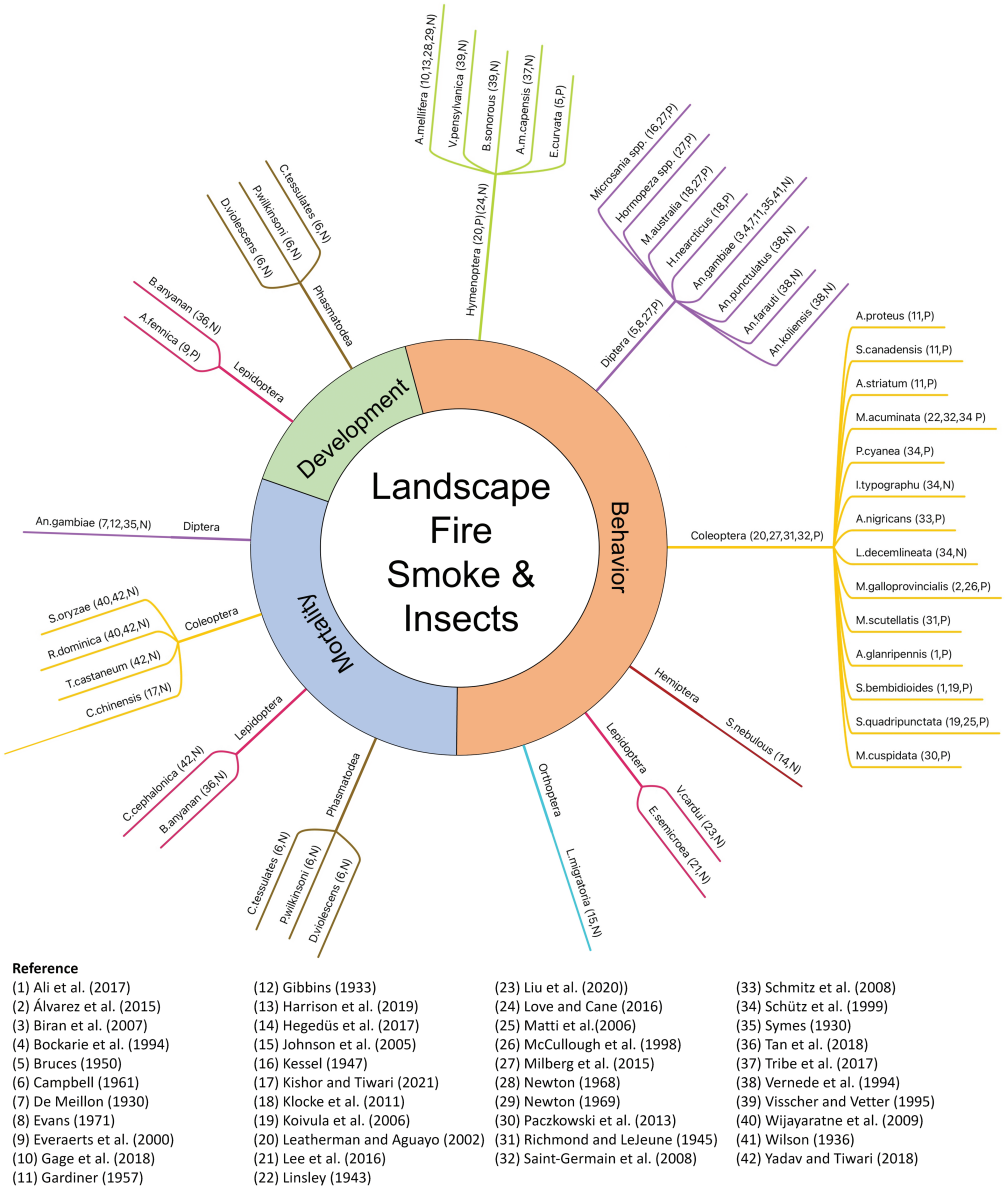
Insect species were studied concerning smoke from landscape fire activities within the published literature. The smoke-related impacts from landscape fire activities on insects have been divided into three aspects, including development, behavior, and mortality. The order and species of insects studied are listed. P refers to Positive impacts and N refers to Negative impacts.

The impacts of smoke pollution on insects recorded in the published literature were divided into three broad aspects: (1) larval development (3 studies); (2) behavior (33 studies); and (3) mortality (6 studies) (Fig. 3). From the literature, four species of insects were demonstrably impacted by smoke across more than one aspect (e.g., Lepidoptera (Family: Noctuidae) had smoke-related impacts on larval development, behavior, and mortality). Three insect orders had evidence of results from only one part: e.g., Hemiptera, Orthoptera, and Hymenoptera were affected in their behavior. The overall findings are summarized below.

#### Impacts on Insect Larval Development

Smoke pollution from burning activities can affect insect growth and lifespan, with one relevant study reporting positive, and two reporting negative influences. Firstly, the larval development of some insect species can be affected. Forest fire smoke was associated with over 90% of eggs of three stick insect species (*Ctenomorphodes tessulatus* [Phasmatodea: Phasmatidae], *Podacanthus wilkinsoni* [Phasmatodea: Phasmatidae], and *Didymuria violescens* [Phasmatodea: Phasmatidae]) failing to hatch (Campbell 1961). Tan et al. (2018) demonstrated that *Bicyclus anynana* (Lepidoptera: Nymphalidae) larvae exposed to burning incense coil simulating forest fire smoke of average PM_2.5_ concentration at 120 µg m^−3^ exhibited significantly decreased survival, prolonged larval development time, and reduced pupal weight compared with those exposed to an average PM_2.5_ concentration of 50 µg m^−3^ (control treatment). An increase in development time and a decrease in pupal weight were also observed when larvae were fed on corn plants exposed to the same concentration of smoke, demonstrating an indirect impact of exposure to smoke. Nevertheless, not all smoke-related impacts are harmful, and some insects respond positively to LFS. The volatiles from burned vegetation stimulated the biosynthesis of virgin female juvenile hormones of *Actebia fennica* (Lepidoptera: Noctuidae), which would accelerate sexual maturation and reduce the period of mating and oviposition. The average number of chorionic eggs in smoke-exposed females was twice that of the control group (Everaerts et al. 2000).

#### Impacts on Insect Behavior

In addition to directly affecting the growth of insects, smoke also impacts insect behavior. Insects may be attracted to, or repelled by, potential substratum-rich recently burned habitats due to the smoke and heat (Evans 1972;, Saint-Germain et al. 2008, Tribe et al. 2017). Fire-favoring beetles, such as *Buprestidae* (Coleoptera) and *Cerambycidae* (Coleoptera), were attracted to fire activity by smoke and reproduced quickly in the freshly-burned forest (Linsley 1943, Gardiner 1957, Ross 1960, Evans 1972, Leatherman and Aguayo 2002, Koivula et al. 2006, Saint-Germain et al. 2008, Paczkowski et al. 2013, Álvarez et al. 2015, Milberg et al. 2015, Elia et al. 2016, Ali et al. 2017). The smoke plumes can attract these insects (Saint-Germain et al. 2008) to habitats where landscape fires are still occurring or have just ended (Richmond and Lejeune 1945, McCullough et al. 1998, Leatherman and Aguayo 2002, Koivula and Spence 2006, Koivula et al. 2006). Guaiacol derivatives released from burning *Pinus sylvestris* (Scots pine) can stimulate the antennae of Jewel beetles (Coleoptera: Buprestidae) (Schütz et al. 1999, Paczkowski et al. 2013). For instance, *Melanophila acuminate* (Coleoptera: Buprestidae) are highly sensitive to guaiacol (Schütz et al. 1999), but *Phaenops cyanea* (Coleoptera: Buprestidae) and *Ips typographus* (Coleoptera: Curculionidae) are less so, although they show escape behavior regarding LFS (Schütz et al. 1999, Schmitz et al. 2000, Álvarez et al. 2015, Ali et al. 2017). More specifically, there are several types of sensilla on the antenna, among which the basal sensilla respond to odor stimulation (Ali et al. 2017). A group of nine cells in *Monochamus galloprovincialis* (Coleoptera: Cerambycidae) was sensitive to smoke plumes which helped them to detect smoke from several kilometers away (Álvarez et al. 2015).

Smoke as a product of fire has also been observed to attract fire-favoring flies, such as *Hormopeza* spp. (Diptera: Empididae) and *Microsania* spp. (Diptera: Platypezidae) (Kessel 1947, Brues 1950, Evans 1972, Leatherman and Aguayo 2002, Milberg et al. 2015). It was reported that dozens of Microsania flies (Diptera: Platypezidae) aggregated and swarmed within the smoke plume due to forest fires (Klocke et al. 2011a, Milberg et al. 2015). In addition to smoke from fires, impacts can also be seen from ‘cold smoke’ sources, for example, an aerosol-bomb-dispensed smoke concentrate used by beekeepers, can also attract *Microsania* spp. (Kessel 1960). Moreover, the burnt sites after a fire can also attract *Hypocerides nearcticus* (Diptera: Phoridae) aggregating and swarming outside the smoke plumes (Klocke et al. 2011a).

Smoke generated from wildfires has also been shown to affect Hymenoptera, including indirectly through habitant change, or directly through injury or death (Love and Cane 2019). Brues (1950) observed that *Eumenes curvata* (Hymenoptera: Vespidae) were attracted to smoke generated from burning weeds, lingering in smoke as they moved back and forth from their nests. However, more studies have observed that smoke could restrain honey bees for an extended period. For instance, *Apis mellifera capensis* (Hymenoptera: Apidae) as a subspecies of *Apis mellifera* (Hymenoptera: Apidae) have been observed to stay far away from their nests because they are sensitive to fire smoke and have continuous absconding behavior (Tribe et al. 2017). This may be because smoke blocks their chemical communication that is needed to coordinate swarming, in particular through weakening of the electroantennography response of their antennae to alarm pheromones (Visscher et al. 1995).

Honey bees whose sensory perceptions were blocked by smoke exposure performed apparent and temporary suppression of aggression compared to those allowed to recognize typical social cues (Harrison et al. 2019). Smoke can affect whether droplets of venom are released with the stinger, although it may have no impact on the likelihood of the sting extending. Smoke from burlap and hops has also been shown to reduce droplet formation and possibly lead to fewer alarm pheromones being released (Gage et al. 2018). When *Apis mellifera* were exposed to smoke, the bees in the colony became engorged (Newton 1968). Smoke also reduces the number of guards and foragers due to the alarm pheromone isopentyl acetate (Newton 1969). Extending the impact to other species, the number of attacks by *Bombus sonorous* (Say) (Hymenoptera: Apidae) and *Vespula pensylvanica* (Saussure) (Hymenoptera: Vespidae) reduced by over two- and ten-fold respectively when smoke was close to their colonies (Visscher and Vetter 1995).

Lepidoptera are also affected by smoke; *Exyra semicrocea* (Lepidoptera: Noctuidae) initiate flight in response to smoke from a periodic fire in pine savannas (Lee et al. 2016). The flight performance of *Vanessa cardui* (Lepidoptera: Nymphalidae) was significantly affected by smoke-contaminated air showing that dense smoke conditions negatively impact the flight performance of the butterfly (Liu et al. 2021).

When smoke from large forest fires darkened the sky, some insects such as grasshoppers and seed bugs decreased their flight distances or delayed their flights/migrations until the weather cleared (Johnson et al. 2005, Hegedüs et al. 2007).

Moreover, smoke from burning domestic fuels has been found to show repellent effects on *Anopheles gambiae* (Diptera: Culicidae) in some developing countries (De Meillon 1930, Symes 1930, Gibbins 1933, Wilson 1936, Bockarie et al. 1994, Biran et al. 2007). It has also been discussed that smoke can have a series of effects on mosquitoes, including deterrence, expellence, reduced abilities to find hosts and bite, knockdown, and death (Vernède et al. 1994).

#### Impacts on Insect Mortality

Smoke can impact insect dynamics at individual and population levels by affecting their growth and behavior and even directly determining their mortality. Over 80% of *Bicyclus anynana* larvae and pupae could not survive in the presence of smoke from incense coil burning (Tan et al. 2018). Smoke from wildfires can also cause bee mortality (Love and Cane 2019). Smoke created from burning cow dung and neem leaves accounted for the high mortality of some Coleoptera species, including *Rhyzopertha dominica* (Coleoptera: Bostrichidae), *Sitophilus oryzae* (Coleoptera: Curculionidae), *Tribolium castaneum* (Coleoptera: Tenebrionidae), and *Callosobruchus chinensis* (Coleoptera: Chrysomelidae) (Yadav and Tiwari 2018, Kishor and Tiwari 2021). In addition, smoke, at a concentration where CO exceeded 5000 ppm, generated by the combustion of dried harvested paddy, may lead to more than 50% deaths of *Rhyzopertha dominica* and *Sitophilus oryzae* when those insects were in a sealed environment for up to 72 hr (Wijayaratne et al. 2009). Furthermore, there was nearly 70% mortality when adult *Corcyra cephalonica* (Lepidoptera: Pyralidae) were exposed to smoke generated from biomass burning for 72 hr (Yadav and Tiwari 2018). However, several early observational studies from Africa showed that the decrease in *Anopheles funestus* (Diptera: Culicidae) does not appear to be caused by the smoke from domestic fires (De Meillon 1930, Symes 1930; Gibbins 1933).

#### Positive or Negative Impacts From LFS on Insects

LFS has both positive and negative impacts on insects. The positive impacts are reflected in LFS attracting insects, especially fire-loving insects, mainly from four insect orders, including Coleoptera, Diptera, Hemiptera, and Lepidoptera (Fig. 3). Some fire-loving insects typically rely on forest fires to reproduce, especially, pyrophilous beetles. They quickly approach persistent fires using antenna sensors to detect the smoke and locate hot spots using infrared radiation sensors, generally located on the thorax or abdomen. Both sensors help pyrophilous beetles to find burning areas. Once they arrive, they can occupy these burnt areas immediately after the fire (for example, Leatherman and Aguayo 2002, Milberg et al. 2015). These pyrophilous insects find suitable habitats by detecting smoke plumes and breeding in these areas, increasing their population.

LFS restricts insect development and repels insects, that are regarded as negative impacts. LFS can inhibit butterfly growth and cause their mortality (for example, Tan et al. 2018). The smoke keeps bees away by disturbing their sense of smell. LFS can suppress the alarm pheromones secreted by bees, and if LFS is sensed by bees, they can be driven to leave their current habitat (for example, Tribe et al. 2017). Sometimes, LFS induces anomalous sky polarization, in which LFS causes reddish skylight, and the degree of linear polarization between skylight and the sun is less than 90°, which can disorient insects (Hegedüs et al. 2007). Moreover, LFS can repel the insects by acting as a camouflage for the signals emitted by the host plant, and insects are sensitive to chemicals in the smoke (Vernède et al. 1994).

### Geographical Distribution of Studies

**Fig. 4.**
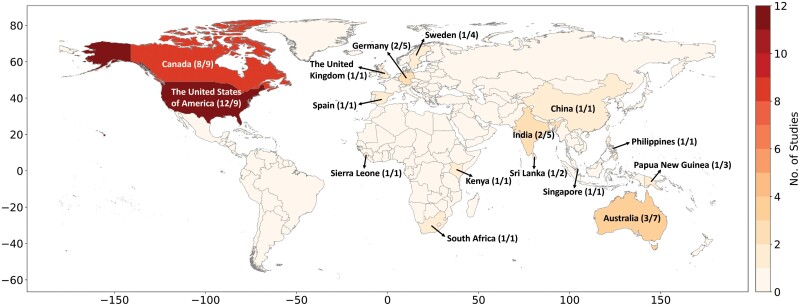
The global distribution of 38 out of 42 smoke-insect studies as determined from a systematic mapping exercise, covering 16 countries, with the number of studies and insect species per country in brackets.

Of the 42 studies reviewed here, 41 studies were conducted in 6 continents, including North America (20 studies), Asia (6 studies), Europe (5 studies), Africa (6 studies), and Australia (4 studies). The remaining 1 study only referred to ‘developing countries’ rather than specific locations. Thirty-eight articles were associated with 16 countries (Fig. 4). Most of the research took place in the United States of America (12 studies), Canada (8 studies), Australia (3 studies), Germany (2 studies), and India (2 studies), with only 1 study in each country of the remaining 12 countries, including Kenya, Sierra Leone, South Africa, China, Singapore, Sri Lanka, Philippines, Papua New Guinea, Spain, Sweden, and the United Kingdom.

The findings are transferable to other locations for some studies involving lab work. For example, lab work conducted in the United Kingdom by Liu et al. (2021), investigating the impact of smoke on butterflies in a combustion chamber, could be applied to various geographical contexts. While these are the countries where impacts are well known, they are not necessarily the countries experiencing the most severe effects from smoke. This is because the relevant research articles on smoke-insect interactions are relatively limited. We did not consider the effect of mosquito coil smoke on insects for the reasons given earlier, however, there are many studies on the repellent effect of household mosquito coils on mosquitoes in Southern Asia (Liu et al. 2003, 2013; Hamid et al. 2017; Amelia-Yap et al. 2018).

The number of specific species studied worldwide is summarized in Fig. 4. The species in those papers investigating the effects of smoke on insects are mainly from the United States of America (9 species), Canada (9 species), Australia (7 species), Germany (5 species) and India (5 species). Three Diptera species and one Coleoptera species were studied in Sweden (Milberg et al. 2015). Three Diptera species were mentioned in Papua New Guinea (Vernède et al. 1994), and two Coleoptera species were studied in Sri Lanka. The remaining eight countries all cover only one insect species.

**Fig. 5.**
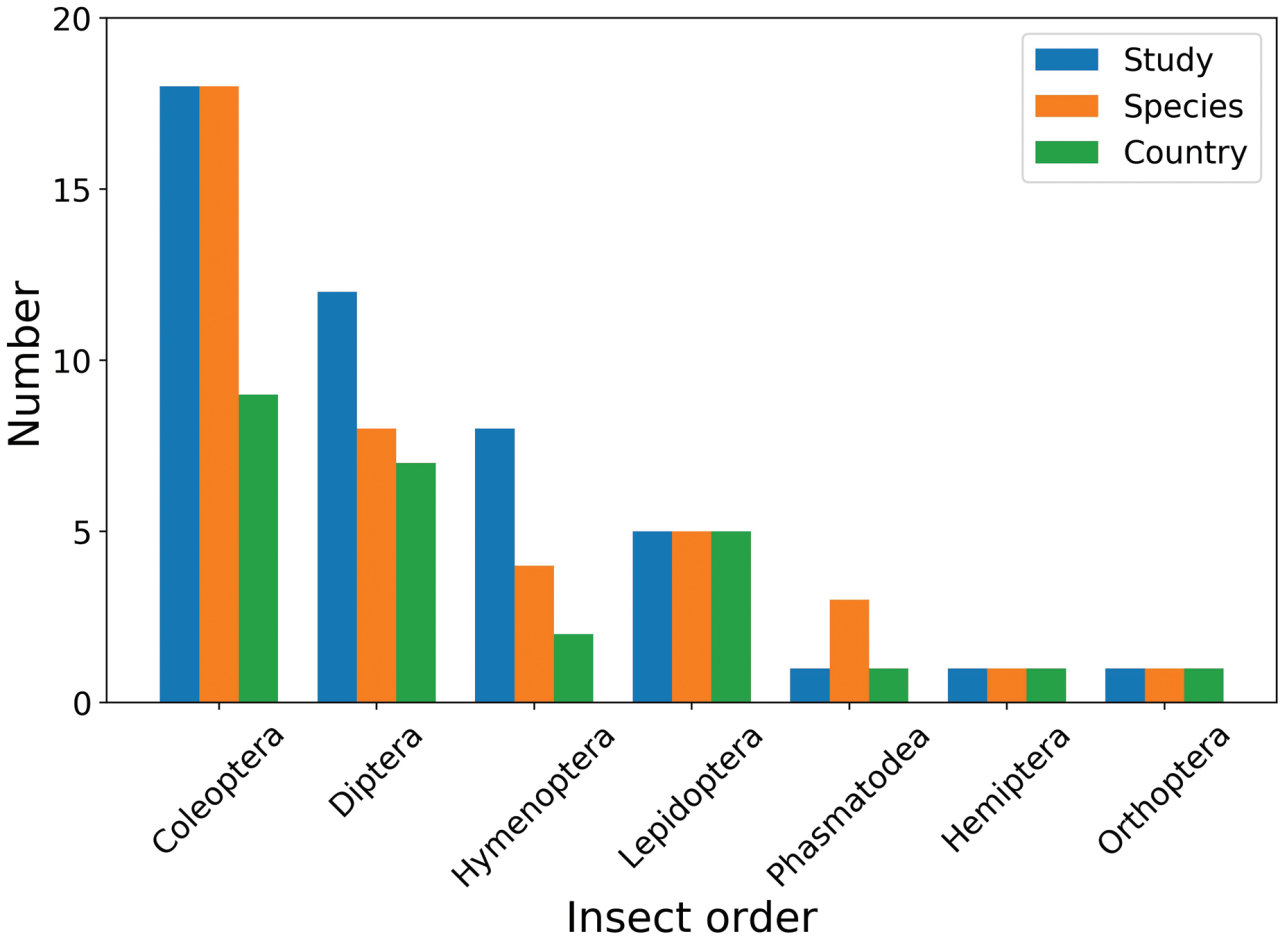
Total number of species, the number of studies, and the number of countries included in the seven insect orders.

The number of specific species, the number of studies, and the number of distributed countries related to the nine known insect species mentioned previously are summarized in Fig. 5. Research on Coleoptera (18 species) was the most prevalent, with 18 studies in 9 countries, including the United States of America, Canada, Germany, Australia, China, India, Spain, Sweden, Sri Lanka. Hymenoptera studies were distributed in the United States of America and South Africa. Diptera studies had a wide research range, including Australia, Philippines, Sweden, and the United States of America, Papua New Guinea, Kenya, and some south and east African areas. Lepidoptera studies were distributed in the United States of America, Canada, India, Singapore the United Kingdom. The Hemiptera study and Orthoptera study were in Canada. Phasmatodea were only studied in Australia. The number of studies did not precisely match the number of insect species investigated in each country because some studies covered more than one insect species, and several papers covered the same species.

## Discussion

Evidence suggests that LFS exposure impacts insect population dynamics through development, behavior, and mortality, although the literature is limited. Approximately 80% of studies discussed how insect behavior responds to LFS, mainly reflected in mating and flight (Hegedüs et al. 2007, Bazzett 2008, Schmitz et al. 2008). These behaviors comprise an extensive range of activities and can eventually affect many aspects including population viability, species persistence, and so on, as described in Lester et al. (2007) and Berg et al. (2010). Understanding the behavior of insects could improve both pest management and conservation programs (Cunningham et al. 1999, Witzgall et al. 2010), and therefore is particularly relevant to human health (e.g., mosquitoes; Greenberg 2019, Steven et al. 2020) and economics (e.g., honeybees; Smith et al. 2013).

### Main Insect Species Studied

In the LFS related articles, we found that three insect groups were most discussed and studied. The most commonly occurring insects in the literature were beetles (Order: Coleoptera), which can be abundant in wildfire areas. Various fire-loving beetles can inhabit burning or burned trees by detecting smoke and heat (Schmitz et al. 2008, Klocke et al. 2011b, Álvarez et al. 2015). For instance, woodboring beetles (e.g., *Buprestidae* and *Cerambycidae*) that regard dead trees as habitat, have a high reproduction rate leading to impacts in their population dynamics (Saint-Germain et al. 2008). Moreover, the population of adult buprestids is found to be higher when burn severity is higher (Ray et al. 2019). Outbreaks of beetles are common in the areas of the U.S. and Canada where wildfires occur frequently due to the increasingly severe drought (McCullough et al. 1998, Gillett et al. 2004, Gavin et al. 2007, Marlon et al. 2012, Ray et al. 2019).

The second group of insects is related to economic activities, such as honeybees (Order: Hymenoptera), which are highly valued worldwide for not only producing honey and wax but also pollinating many crops (Sabbahi et al. 2005, vanEngelsdorp and Meixner 2010, Smart et al. 2016). Smoke can reduce the aggression of bees and is therefore used as the most basic and effective method to obtain honey (Crane 1983). Moreover, bees are sensitive to smoke, so bee behavior may be used to predict the impact of LFS on their colony, particularly in forested areas (Moretti et al. 2009, Galbraith et al. 2019).

The third group of studies focused on those insects having a direct relationship to human health, such as flies and mosquitoes (Order: Diptera), through their spread of diseases and their being of general public health concern (Harrison 1979, Beier 1998, Lacroix et al. 2005, Peter et al. 2005, Vijay Kumar and Ramaiah 2008, Greenberg 2019, Steven et al. 2020). For example, *Drosophila melanogaster* has been commonly used as a research model for human diseases because it is a widely studied and efficiently handled genetic model organism (Kale and Baum 1982, Hamatake et al. 2009, Yamaguchi and Yoshida 2018, Santalla et al. 2021). *Anopheles gambiae* has been studied for decades because it spreads malaria. Smoke from burning plants or wood is often used to repel mosquitoes (Vernède et al. 1994), and therefore, information on their efficacy is vital in a public health context.

### Main Smoke Sources Studied

LFS comes from various natural sources, but most attention is given to wildfires, particularly forest fires, while others include wood fires and prescribed fires. Fires and insects work interactively as the disturbance agents to the ecosystems of many forests, which have effects on the composition of the species in the forests (McCullough et al. 1998, Swengel 2001). Prescribed fires are primarily used as a land management tool to control the natural fires and reduce the frequency or severity of wildfires (Ryan et al. 2013). Swengel (2001) summarized how insects responded to fires, including wildfire and prescribed fires. Several pieces of evidence showed that local ecosystems would be maintained and improved by managed fires because they are controlled and can have positive impacts on biodiversity while wildfire is normally uncontrolled, irregular, and damaging (Ferrenberg et al. 2006, Fernandes et al. 2013). Hence, some insects associated with herbaceous vegetation responded favorably. For instance, the exacerbated landscape fires induced by the El Niño Southern Oscillation (ENSO) event of 1997-1998 in East Kalimantan, via its ability to depress rainfall, caused approximately 90% of forest cover over a 400 km^2^ area in the Balikpapan-Samarinda region to burn (Harrison 2000, Cleary and Grill 2004). Although many insect species significantly declined following this event, the proportion of *Jamides celeno* (Lepidoptera: Lycaenidae) increased from less than 5% in the pre-ENSO butterfly assemblage to over 50% in the post-ENSO assemblage, becoming the dominant butterfly species in the local area (Cleary and Grill 2004). However, whether wildfire or prescribed fires, the smoke emissions are similar, and the concentration of the emitted substances depends on the proximity to the source of the fire (Navarro et al. 2018). In the related articles studying the impacts of LFS on insects, it is hard to collect accurate information on wildfire density, length, and area because researchers have predominantly focused on how insects respond to smoke from forest fires rather than studying the fires themselves. Some observations were over a short period (e.g., five days, Richmond and Lejeune 1945; Johnson et al. 2005), while some were studied over a far more extended period (e.g., 30 yr, Saint-Germain et al. 2008). One driver of this lack of study may be that fire-loving insects are difficult to sample, except after a bushfire (Milberg et al. 2015). Most of the articles qualitatively described the observed smoke conditions or weather changes caused by the smoke, and a few articles quantified the concentration of the gases and PM_2.5_ to specify the severity of the smoke conditions (Wijayaratne et al. 2009, Tan et al. 2018, Liu et al. 2021).

### Main Locations Studied

The distribution of insect studies broadly follows trends in global wildfire distribution, particularly for field-based studies. Lu et al. (2021) studied global fire distribution using remote sensing data (VIIRS 750m) and showed that high-frequency fires are distributed in North America, Australia, and Africa. The historical focus on these geographical regions is understandable. However, it is still somewhat surprising that there is so little focus on the ecological impacts of smoke on insects, particularly given (1) their critical functional roles in ecosystems globally (Humphrey et al. 1999), (2) the global diversity of insects (Gaston 1991), (3) the increasing and dramatic ecological impacts that may result from changing wildfire regimes in some regions (McKenzie et al. 2014), and (4) the extensive entomological literature that covers many thousands of species. Indeed, the relationships between insects and smoke need further investigation. Most published research has focused on impacts on insect behavior and larval development and to a lesser extent mortality. There is a gap for more research, particularly into smoke impacts on population ecology and how this may influence community composition and diversity.

### Other Fire Factors Impacting Insects

In addition to LFS, other fire factors may also influence the insect community. Koltz et al. (2018) summarized the direct and indirect impacts of fire factors on insects, such as fire intensity, frequency, and severity. These fire factors may impact insect dispersal ability, life stages, diet, and habitat utilization. Fire at high frequency but low severity may attract fire-loving insects and increase insect diversity, while fire at high frequency and high severity may negatively impact insect recovery (New 2014). Fire at high frequency but low intensity can significantly reduce some insect numbers, such as beetles and bugs (York 1999). Swengel (2001) illustrated that insect species abundance significantly reduced after a fire, which can be an effective conservation management tool for open habitats. The magnitude of the reduction in insect populations was related to flame exposure. However, insect species diversity can be increased due to frequent prescribed fires by controlling the local plants and maintaining habitats (Ferrenberg et al. 2006, Ulyshen et al. 2021). For instance, the species richness of saproxylic beetles increased after a prescribed fire compared to a set of unburned sites (Ulyshen et al. 2020). As Kral et al. (2017) show, the response of different species of insects to fire is variable, not simply an increase or decline, and is driven by multiple factors.

### Climate Change Impacts on LFS

Changes in fire frequency, intensity, and severity lead to potential impacts on the ecology of a region (Dale et al. 2001). Climate change increases the temperature globally and changes precipitation patterns, which aggravates frequency, severity, and extent of landscape fire activities (Moritz et al. 2012, Pachauri et al. 2014, Reid et al. 2016). Dupuy et al. (2020) indicated that the probability of wildfires in Europe increases by 2% this century due to climate change, while the burned area is likely to increase by 45%. This increase in wildfires could result in severe atmospheric pollution both locally, and globally (Reid and Maestas 2019). For instance, Indonesian forest fires have been shown to impact air pollution in the neighboring country of Singapore (Sheldon and Sankaran 2017). The increase in the burned area could lead to a doubling of the current carbonaceous aerosol emissions from wildfires by 2050 (Spracklen et al. 2009). These fire smoke impacts may be seen in the structure and function of insect communities (Koltz et al. 2018).

### Recommendations for Future Research

Most behavioral work has examined how smoke (and burning more generally) may attract or repel insects, with some biochemical work on response mechanisms. As insects have different functional roles within ecosystems, such as plant pollinators (Ollerton 2017) and seed dispersers (Farwig and Berens 2012), one potential research area is the impact of LFS on insect ecosystem services. Sagili and Chakrabarti (2021) suggested that smoke pollution from wildfires decreased the pollination services of honey bees, providing rare evidence showing that LFS impacts insect ecological function. We suggest that the impact of various types, concentrations, and compositions of smoke emission on ecosystem service aspects of insect ecology should be priorities for future research.

Another potential direction is studying the flight behavior of insects under smoke conditions, especially as many impacted functions of insects relate to their flight behavior. Apart from some recent work on butterflies (Liu et al. 2021), there is little work on how smoke may impact the flight behavior of insects, including flight initiation, speed, duration and flight direction-finding ability. It is necessary to explore their flight performance under different LFS conditions. Conditions in the atmospheric environment can substantially impact insect migration, one of which is that insect migration usually occurs on clear days (Drake and Farrow 1988). LFS can cause extreme weather phenomena that are detrimental to insect migration. If insects do not migrate, they are likely to be trapped in the fire-prone region, ultimately threatening survival (Hegedüs et al. 2007).

In addition, it would be important to investigate how smoke emissions may impact insect reproductive behavior (Ridley 1988, Musolin 2007). As Tan et al. (2018) have shown, not only can smoke pollution negatively impact butterfly development, but also reproductive capacity and behavior. Some fire-loving insects complete their reproduction in burning trees during forest fires (Schmitz et al. 2008). Usually, plant succession after landscape fires creates habitats for various insects, which causes insect outbreaks (Sanderfoot et al. 2021). For instance, the abundant resprouting of host plants after a fire provides a habitat for butterflies, allowing their populations to increase (Cleary and Grill 2004). When LFS impacts insect population distribution and habitat, it may impact insect metapopulations at a broad spatio-temporal scale, where effects are seen among interacting insect populations (Singer and Wee 2005), including population genetics (Nyabuga et al. 2012). The environmental change also impacts the structure of insect assemblages, although the correlation between environmental variables and assemblage structure is relatively weak (Heino and Mykrä 2008). Knowledge of these areas is essential not just for understanding the ecology of insects but also for the ecosystem services that are associated with them because insects are crucial components of biodiversity in most terrestrial ecosystems – as predators, parasites, herbivores, saprophages, and pollinators (Schowalter 2016, Brockerhoff and Liebhold 2017). More evidence is needed to determine what specific components in LFS impact insects to better predict insect performance under various degrees of atmospheric pollution.

Furthermore, the responses of insects to different sources of smoke pollution can be studied and summarized. For example, when *Drosophila melanogaster* are exposed to cigarette smoke for over six hours, there is an increasing possibility of gene mutagenesis (Uchiyama et al. 2016). Some butterfly species can display mortality when exposed to high-level air pollution induced by coal power plants, like *Thecla betulae* (Lepidoptera: Lycaenidae) (Corke 1999). Although the chemical components in cigarette smoke, power plant emissions, and LFS are different, similar impacts may (or may not) result. More specifically, the concentrations of gas and particulates in the smoke need to be measured in the future study. There were some pieces of evidence showing that specific gas component impacts insects. For instance, *Drosophila melanogaster* exposed to SO_2_ concentrations at 400 mg m^−3^ significantly decreased pupal survival and adult endurance in the polluted environment (Ginevan and Lane 1978). Also, this was observed for the larvae of *Junonia coenia* (Lepidoptera: Nymphalidae), when reared under high CO_2_ conditions (700 mg m^−3^) grew significantly slower and took longer to pupate compared to those larvae in ambient CO_2_ conditions (300 mg m^−3^) (Fajer et al. 1991). Although some studies measured PM_2.5_ concentration to show how severe the smoke conditions, such as Tan et al. (2018) and Liu et al. (2021), many LFS-insects studies did not describe the specific components in the smoke.

To achieve what has been mentioned above, both field work and laboratory experiments are required to enable controlled conditions and allow target organisms to react more naturally to smoke in their environment. Specific measurements of smoke characteristics can be challenging, but as far as possible, this should be conducted in future work to increase comparability and transferability of results. It was not always possible to ascertain key smoke characteristics from some of the studies reviewed here, for example, smoke concentrations or components. Many papers considered only general smoke from wildfires, meaning that while the impacts may be clear, in the absence of details on concentrations and components, the key drivers of the impact may not become apparent. An essential aspect of future research will be the more significant investigation of smoke components from different sources, including different types of wildfires, controlled agricultural burning, and domestic sources (Sun et al. 2014, Tan et al. 2018).

## Conclusions

We reviewed the effects of LFS on insects and summarized the information identified. LFS can be used as a cue to attract insects who ultimately find suitable habitats, such as fire-loving beetles (Saint-Germain et al. 2008). However, it can also trap or repel insects, such as honey bees (Tribe et al. 2017). Besides the impact on insect behavior, LFS can also inhibit insect development and cause mortality, for example, in butterflies (Tan et al. 2018) and moths (Yadav and Tiwari 2018). Most studies relating to LFS effects on insects have concentrated on developed countries, though landscape fire activity is highest in developing countries and regions. More information is needed in these areas to develop a comprehensive understanding of ecological feedback in response to LFS, such as in regions of Southern Africa, South Asia, and South America. So far, only seven orders have been studied concerning the effects of LFS. Therefore, a wider range of insects need to be taken into consideration to understand the broader effects of LFS and enable these impacts to be considered when attempting to understand the future impacts of landscape fires under changing climate and human activity.
